# Current knowledge and scientific trends in myokines and exercise research in the context of obesity

**DOI:** 10.3389/fmed.2024.1421962

**Published:** 2024-09-23

**Authors:** Austėja Letukienė, Vaiva Hendrixson, Valentina Ginevičienė

**Affiliations:** Faculty of Medicine, Vilnius University, Vilnius, Lithuania

**Keywords:** myokines, physical activity, obesity, VOSviewer, bibliometric analysis

## Abstract

The relationship between exercise and obesity has attracted increasing attention from researchers worldwide in recent years. The aim of the present study was to analyze the current knowledge and scientific trends of research into myokines and exercise in the context of obesity and provide ideas for future research strategies to prevent obesity. The study conducted a comprehensive bibliometric analysis of 300 scientific publications related to myokines, exercise, and obesity from 2004 to 2024. Applying the VOSviewer tool, the analysis revealed a significant increase over time in the number of publications on these topics, with a total of 1,142 related keywords identified. Key themes identified in the analysis included molecular processes, new organokines, skeletal muscle research, model organism studies, and human studies based on sex and age differences. The study highlighted the growing interest in the molecular mechanisms of obesity and role of myokines. Results showed a substantial increase in publications from 2014 to 2024, with a focus on new organokines (myokines, adipokines) and animal models. The analysis underscored the importance of myokines in modulating metabolic processes and their potential therapeutic implications in managing non-communicable diseases such as obesity. Furthermore, the study revealed the close relationship between exercise, myokine production, and regulation of metabolism, stress response, and inflammation. In conclusion, over the last years, increasing research interest has been focused on the molecular mechanisms of obesity and benefits of exercise, and probably will be focused on a set of myokines released during muscle contraction. A newly identified myokines has emerged as a promising marker for the prevention and control of obesity.

## 1 Introduction

Obesity is a heterogeneous, complex, and multifactorial disease diagnosed when body mass index (BMI) exceeds 30 kg/m^2^. Estimates vary, but twin, family and adoption studies show heritability rates of BMI between 40 and 70%. The most common is polygenic obesity (about 95% of the obese individuals), which results from interactions between genetic and environmental factors. Highly penetrant are syndromic and monogenic forms of obesity result from rare genetic variants and minimal environmental factors. Obesity can affect individuals of all ages, sexes, and ethnicities (1–3). The main causes of polygenic obesity are associated with lifestyle, namely physical inactivity and energy intake exceeding requirements. When the energy balance is disrupted and excess fat tissue accumulates due to obesity, molecular processes and signaling pathways regulating metabolism, appetite, inflammatory responses, and other processes undergo changes (3, 4). The main methods of treating obesity currently involve lifestyle modifications, including exercise training and balanced nutrition (2, 5). The relationship between exercise and obesity has attracted increasing attention from researchers worldwide in recent years. Scientists hypothesize that physical inactivity and sedentary lifestyles contribute to excess adipose tissue and the development of inflammatory processes, while physical activity and regular exercise suppress inflammatory reactions, increase metabolism, and reduce BMI (2, 6). It has been found that exercise helps not only in controlling body weight but also in improving health and reducing the risk of obesity-related diseases such as cardiovascular diseases, type 2 diabetes, hypertension, and atherosclerosis (6, 7). Researchers worldwide are currently making significant efforts to understand the homeostatic regulation mechanisms of a healthy and obese individuals by studying the interaction between tissues and cells, as well as newly discovered circulating factors and cytokines known as organokines (such as myokines, hepatokines, and adipokines) (6, 8–10). Muscles, the liver, and adipose tissues produce and secrete specific organokines in response to environmental stimuli (physical activity, nutrition, etc.). Organokines are crucial factors in interorgan crosstalk. Recent evidence suggests that dysregulation of the interplay of organokines between organs is associated with the pathophysiology of obesity (10, 11).

Recent research suggests that myokines are muscle-derived signaling proteins that cause humoral changes in the body, acting as hormones and exerting autocrine, endocrine, and paracrine effects in other organs (e.g., adipose tissue, brain, and liver) (6–14). Thus, myokines mediate the communication between muscles and all other systems of the body (6, 12, 14). Myokines perform their positive function only during exercise when the muscles contract and myokine concentration in the blood reaches normal levels (6). Obesity can cause changes in the release level of myokines, such as increased cytokine activity, and disturbances in their mechanisms of action in physically inactive lifestyles (5, 6). Meanwhile, exercise transmits signals for myokine production and release into circulation (6, 10). The production of myokines in muscles depends on muscle activity, intensity, and duration of movement (7, 8, 15). Furthermore, muscle contraction is a necessary condition for activating the function of other stimuli, for example, those involving intracellular and extracellular signaling pathways, and the stimulus effect is observed not only in muscles but also in other organs or tissues (including adipose tissue, the liver, the brain, etc.) (7, 10, 13, 14, 16). However, the molecular mechanism of myokines and inter-organ communication in relation to exercise and disease pathophysiology is still not well understood, and it is unclear which myokines participate in homeostasis and how their expression and interaction occur depending on exercise (i.e., frequency, intensity, duration, and volume). To conduct further research in this area, it is necessary to analyze previous scientific studies and data, as well as relevant information from publications. Bibliometric analysis is therefore the most suitable method to summarize the current research situation and quantitatively predict future research trends. Nevertheless, to our knowledge, no bibliometric studies have assessed myokine, exercise, and obesity research at the global level.

The aim of this study was to analyze the current knowledge and scientific trends of myokines and exercise research in relation to obesity and provide ideas for future research strategies to prevent obesity (based on bibliometric analysis and visualization technology).

## 2 Materials and methods

The research model is based on a quantitative and qualitative analysis study of scientific publications and other scientific documents. We investigated the growth and citation of publications, active authors, countries, and institutions, and the frequency of terms (myokine and exercise studies in the context of obesity) through a bibliometric/scientometric analysis. The bibliometric analysis was performed using a visualization of similarities viewer (VOSviewer version 1.6.20). VOSviewer is a software tool for constructing and visualizing bibliometric networks of meta-data.

Data were obtained from the PubMed database (on February 2024). Ethical approval for this study was not necessary because the data used were obtained from a public database and involved no interaction with human or animal subjects.

### 2.1 Data collection

The data search was focused on the terms myokines, exercise, and obesity and associated terms. The search strategy employed was as follows: the use of the Boolean operator AND: ([exercise] AND [myokine] AND [obesity]). Academic articles published between 2004 (the first publication) and 2024 were filtered using relevant keywords. All the documents were downloaded as “Full Record and Cited References” and saved as “Bibtex” and “plain text file” for further analysis. The meta-data of these publications, encompassing authorship, titles, journal sources, citation counts, publication years, etc., underwent processing within VOSviewer. In order to analyze the changes in research topics and areas over the years, bibliometric analysis was conducted by categorizing scientific publications into the past two decades: the first one from 2004 to 2013 inclusive and the second from 2014 to February 2023.

### 2.2 Data processing, visualization, and analysis

Microsoft Office Excel 2016 (Microsoft Corporation, Redmond, WA, United States) was used to stratify and systematically assess the sorted publications and data. The number of studies was counted by publication year, research area, journal, country of publication, research organization, and author. The files containing data on the topic were imported into the VOSviewer software to perform bibliometric analysis and data visualization. During the analysis, a network visualization map was drawn step by step according to the setting parameters by VOSviewer. We generated network maps of research organizations, authors, and keywords. Clusters of connections (author and collaboration networks, and keyword associations) were formed automatically in this study, and the clustering resolution was appropriately adjusted as needed. The size of the circle nodes in the visualized map showed the frequency of publications or keywords, the link between nodes showed associations (such as co-occurrence), and the distance between nodes showed the degree of association. Typically, a visualization encompasses only one form of linkage. In cluster analysis, different node colors represented different clusters, and depending on the similarity threshold between nodes, the number of clusters varied. The total link strength (TLS) parameter, which indicates the strength of relationships (the sum of the link strengths of nodes), was also evaluated. The TLS was automatically calculated by VOSviewer, where a higher TLS value indicates a greater relationship between keywords. Each link is characterized by its strength, which is denoted by a positive numerical value. Closer placement generally signifies stronger correlation. It helps identify most occurrences of keywords, their associations, popular topics, and authors who extensively study the topic of interest. An element is assigned exclusively to one cluster, and clusters are not obliged to encompass all elements. Consequently, there May be elements not affiliated with any cluster. Clusters are identified using numerical labels, and the color of an element corresponds to its cluster affiliation.

From this analysis, we intended to find popular research topics on myokines and exercise studies in the context of obesity. Keyword and significant word trends encompassing citation relationships among institutions and their researchers were inspected.

## 3 Results

**Figure 1:**
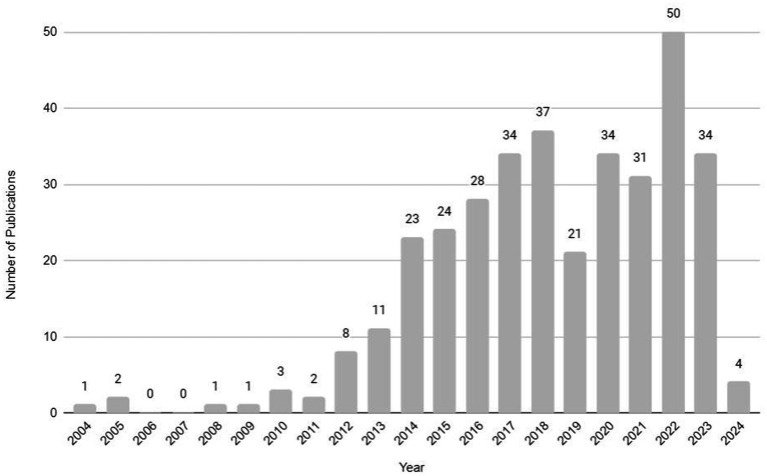
Number of publications about obesity, myokines, and exercise from 2004 to 2024 (source: PubMed database, accessed February 2024).

At the beginning of the study, the main themes of scientific publications related to obesity, exercise, and myokines were analyzed with the use of the VOSviewer bibliometric analysis tool. A search was conducted using the keywords obesity, myokine, and exercise in the PubMed database. The query resulted in a total of 300 articles (which included original research, review articles, case reports, and clinical studies or clinical trials) and were published from 2004 (first publication) to 2024 as shown in Figure 1.

In 2014, there was a significant increase in the number of publications (it doubled), followed by gradual growth. Although there was a sharp decline in 2019, the trends returned to previous levels in the following years, with a second exponential growth in research numbers observed in 2022, indicating escalation in interest. Subsequently, it appears that since 2023 the number of studies has stabilized once again. We expect that beyond 2024 the field of obesity, myokines, and exercise-related research and studies will continue to experience a progressive increase in the number of publications.

A total of 1,637 authors have contributed to the PubMed database at least one publication matching the search criteria for the keywords obesity, exercise, and myokine. One hundred sixty-seven have published two articles, 39 have published three articles, 22 have published four articles, and 8 authors have published five scientific articles related to this topic. Notably, the highest number of publications (*n* = 7) and the highest h-index (154) were attributed to the American scientist Mantzoros from Harvard Medical School in Boston. Additionally, Bilski from Jagiellonian University in Poland and Eckel from the German Diabetes Center demonstrated remarkable activity in this research area, each with six publications. The analysis revealed that the most prolific contributors, those publishing more than five papers on this specific topic, were eight researchers from the USA, Germany, Egypt, and Poland and a group of three individuals from South Korea. Notably, 50% of the research was conducted and published by European scientists, with 35% of that originating from Jagiellonian University in Poland. The second most active region was Asia, representing 35% of the research, with the majority (25%) coming from South Korea.

A total of 919 research organizations have contributed at least one publication. Among them, 6 organizations published up to three articles, 25 organizations provided two articles, and the other 888 organizations submitted only one article each. European universities or medical or research centers in eight different countries produced 43.9% of all publications. Asian universities in three countries accounted for 34.8% of publications. Research groups from South America, Australia, and Africa contributed a smaller number of publications. Examining organizational activity, it is evident that Jagiellonian University, with a global university ranking QS of 339, in Europe (Poland) emerged as the most active (*n* = 8). The second most active is Chung-Ang University, with a global ranking QS of 494, in Asia (South Korea) (*n* = 7). The third position is held by Tecnologico de Monterrey, with a global ranking of 759, in South America (Mexico) (*n* = 6). In summary, active research on this topic in North America was not observed, while European universities, particularly Jagiellonian University, stand out as global leaders. The average global ranking of universities is 443, with the highest position (10th) held by Karolinska Institutet in Sweden. In conclusion, analysis of the focal points and locations of research, along with the likely target groups, suggests that there is still insufficient information about specific populations.

### 3.1 VOSviewer mapping results of keywords network analysis

In the next stage of the study, the most frequently occurring keywords related to myokines and exercise in the context of obesity were used in the network analysis of final keywords (by VOSviewer mapping). Duplicates and insignificant keywords or repetitive concepts were excluded from the final list of keywords.

Analysis of 300 publications from the PubMed database returned 1,142 significant related words/terms (keywords), of which 128 were mentioned in publications at least 5 times. During the first decade of research in this field from the first publication in 2004 to 2013 (inclusive), a total of 46 articles were published, and these were associated with 266 keywords, of which 23 were mentioned at least 5 times. Each publication had an average of 5.8 different connected keywords. During the second decade from 2014 to February 2024, a total of 271 articles associated with 1,058 keywords were published, of which 111 were mentioned at least 5 times. On average, each publication had 3.9 different related keywords. Consequently, during the period from 2014 to 2024, there were 12.5 times more scientific publications on the topic analyzed than there were from 2004 to 2013. There were 4 times more new individual significant words/terms identified.

Since the number of publications significantly increased from 2014 onwards, the distribution of these keywords was evaluated separately, considering their proportion in the overall context of all publications. Thus, in order to analyze the changes in research topics and areas over the years, bibliometric analysis was conducted by categorizing scientific publications into two decades. The results of bibliometric analysis of the most occurring and related keywords (on the topic of myokines and exercise in the context of obesity) based on scientific publications from 2004 to 2013 and from 2014 to 2024 periods are presented in Table 1.

**Table 1:** The list of top ten related keywords/terms on the topic of myokines and exercise in the context of obesity based on scientific publications from the periods 2004 to 2013 and 2014 to 2024 (by VOSviewer mapping).

Most frequent co-occurring terms from 2004 to 2013	Percentage of publications (%)	TLS	Most frequent co-occurring terms from 2014 to 2024	Percentage of publications (%)	TLS
Skeletal Muscle	52.2	202	Skeletal Muscle	33.9	1793
Fibronectins	34.8	145	Fibronectins	32.5	955
Insulin Resistance	28.3	103	Insulin Resistance	21.8	640
Adipokines	23.9	90	Irisin	20.3	517
Interleukin-6	19.6	84	Adipose tissue	14.0	408
Type 2 Diabetes Mellitus	19.6	83	Adipokines	12.9	378
White Adipose Tissue	15.2	73	Type 2 Diabetes Mellitus	12.5	367
Adipose Tissue	21.7	72	Energy Metabolism	10.0	338
Brown Adipose Tissue	13.0	63	Inflammation	11.4	315
Cytokines	17.4	59	Body Mass Index	8.9	286

TLS, total link strength, refers to the overall strength of the connection, where a higher value of this parameter indicates a stronger direct relationship between significant co-occurring words.

It was determined that the first three keywords/terms (skeletal muscle, fibronectins, and insulin resistance) have remained unchanged as primary research topics in both decades (2004–2013 and 2014–2024) and persist as the main focal points of investigation in these thematic areas. Despite being less frequently used, adipokines and type 2 diabetes remain among the top 10 relevant keywords in research. Meanwhile, adipose tissue has become an even more prevalent keyword and remains a research focus. During the first decade, keywords such as white adipose tissue, brown adipose tissue, and cytokines like interleukin-6, were among the most popular. However, in the second decade, these topics were investigated less frequently, and new keywords related to obesity, myokines, and exercise (like irisin, energy metabolism, inflammation, and body mass index) gained prominence.

**Figure 2:**
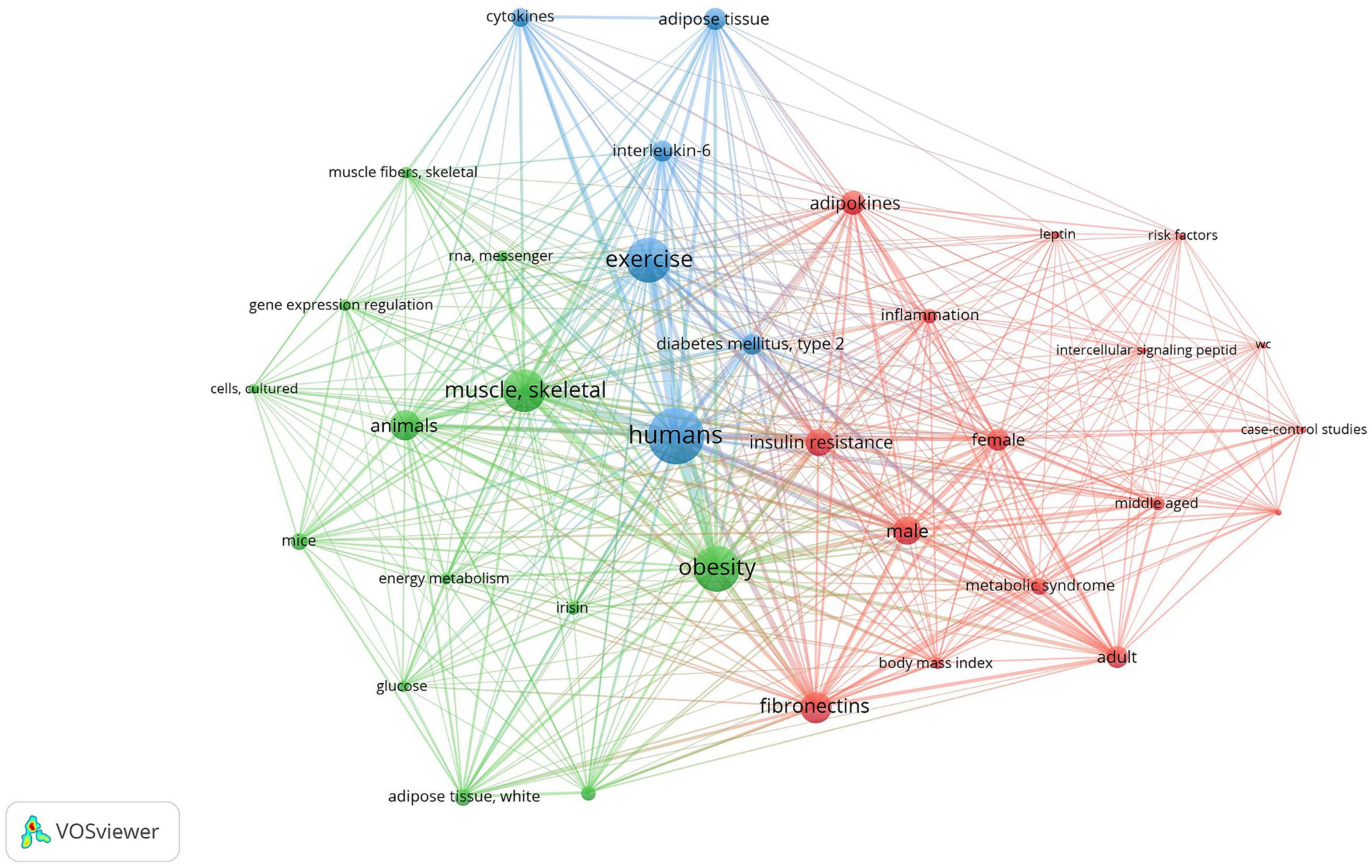
VOSviewer visualization: high-frequency keyword co-occurrence network illustrating the thematic structure of research in obesity, myokines, and exercise based on scientific research in 2004–2013. Nodes represent individual keywords, with node size indicating frequency of occurrence. The lines connecting nodes represent co-occurrence relationships between keywords, with line thickness reflecting the strength of association. Color-coded clusters highlight distinct thematic groups, representing key research areas and their interconnections. This visualization provides insight into emerging trends and primary focus areas within the field. Three clusters are shown on the map and listed on Table 2 and the most frequently used keywords in this research field are listed in Table 1. Source: own study based on data retrieved from the PubMed database in February 2024.

**Figure 3:**
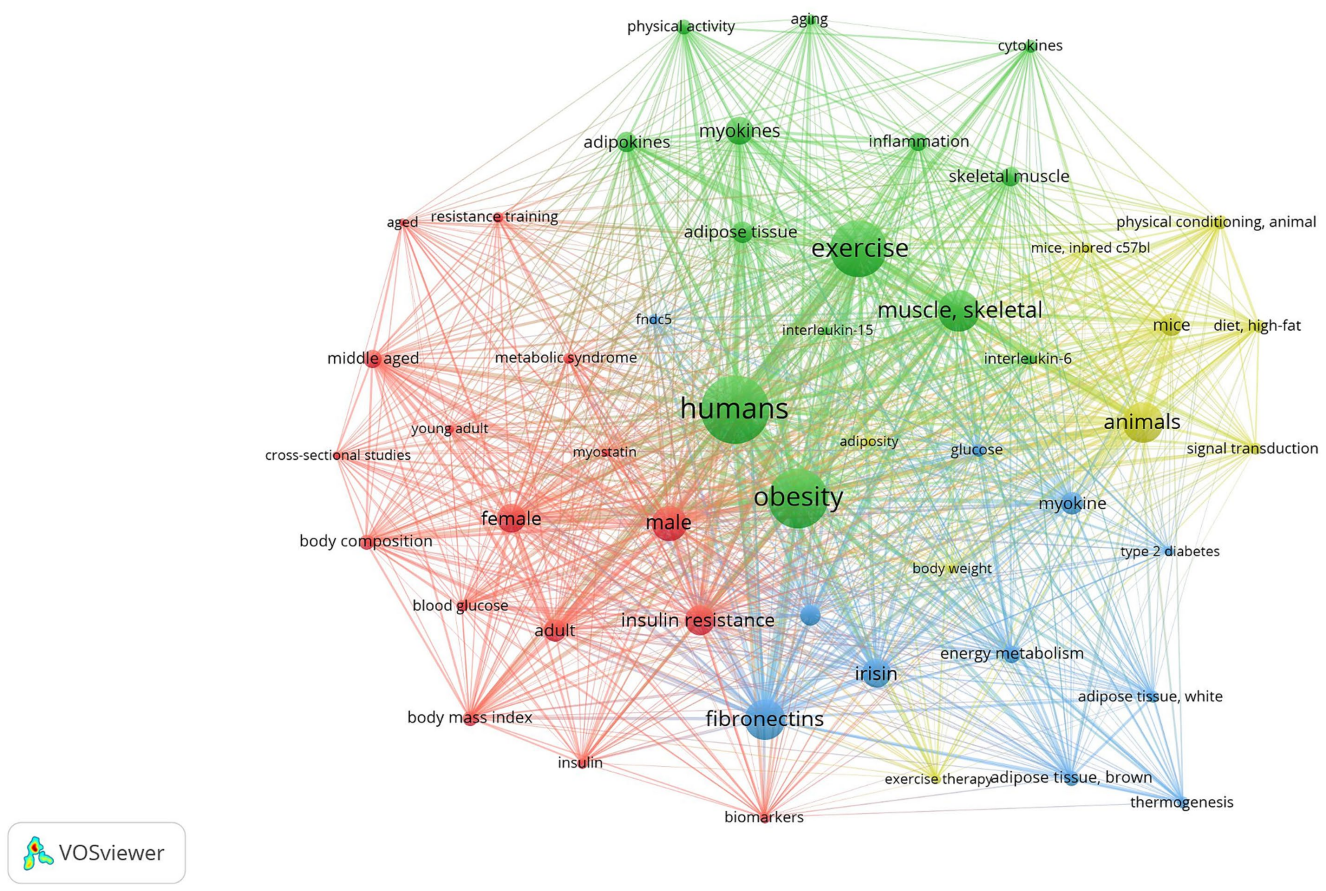
Keyword co-occurrence network depicting the thematic landscape of research in obesity, myokines, and physical activity using VOSviewer. Each node corresponds to a keyword, with node size proportional to its frequency in the dataset. The edges (lines) between nodes represent co-occurrence relationships, with the distance between the lines indicates the overall link strength. Distinct color-coded clusters identify major research themes and their interconnections. This figure facilitates the identification of prevalent research topics and emerging trends within the field. This analysis was performed using studies from 2014 to 2024. The clusters and their associated keywords are listed in Tables 1, 2.

We used VOSviewer for building and visualizing bibliometric networks and for analyzing relations between keywords. The most frequent keywords that were not informative or similar in the context of obesity and exercise were excluded from the analysis. The visualization of bibliometric analysis results of both decades is shown in Figure 2 (2004–2013) and Figure 3 (2014–2024).

The clusters and their constituent keywords formed themes and defined research areas, which depend not only on the frequency evaluation parameter (link) of the repeated keywords but also on the values of the parameter measuring the overall link strength (TLS).

It was found that the three clusters formed in 2004–2013 encompass a wide range of topics (Figure 2):

***Cluster 1*** (blue) is related to a complication of obesity (specifically type 2 diabetes [TLS = 6, 29 links]) and signaling pathway analysis, primarily including adipose tissue (TLS = 7, 21 links) and signaling molecules of the cytokine group (TLS = 5, 19 links), more precisely, interleukin-6 (IL-6, TLS = 5, 29 links), and inflammatory cytokines.
***Cluster 2*** (green) mainly focused on skeletal muscle (TLS = 17, 30 links) and studies of model organisms (animals [TLS = 9, 29 links]; mice [TLS = 3, 22 links]; cultured cells [TLS = 3, 22 links]). Two subthemes stands out: one including gene expression (regulation [TLS = 2, 22 links] and mRNA [TLS = 2, 22 links]), and another one including energy metabolism (TLS = 4, 21 links), focusing on glucose (TLS = 3, 19 links) as a source of energy and white adipose tissue (TLS = 6, 23 links) as accumulated excess energy, as well as myokines like irisin (TLS = 2, 23 links).
***Cluster 3*** (red) essentially focuses on biomarkers (risk factors [TLS = 1, 21 links], adipokines [TLS = 5, 32 links], emphasizing leptin [TLS = 1, 24 links], intercellular signaling peptides [TLS = 2, 21 links], fibronectins [TLS = 12, 32 links]), and complications of obesity: insulin resistance (TLS = 9, 33 links), inflammation (TLS = 4, 31 links), and metabolic syndrome (TLS = 5, 27 links) and the relationship with body mass index (TLS = 4, 27 links). In human research (specifically case–control studies [TLS = 2, 19 links]) the subjects were divided into groups according to sex (male [TLS = 9, 32 links] and female [TLS = 7, 32 links]) and age (middle-aged [TLS = 4, 24 links] and adult [TLS = 8, 28 links]).

In 2014–2023 the thematic scope of clusters narrows and focuses on a four specific scientific topics (clusters) (Figure 3):

***Cluster 1*** (green) represents muscle-adipose tissue interaction studies. This cluster reflects the effects of exercise on skeletal muscle (TLS = 723, 49 links) and myokines secreted by skeletal muscle (TLS = 340, 46 links), adipose tissue (TLS = 311, 47 links) and adipokines secreted by adipose tissue (TLS = 377, 48 links), and cytokines (TLS = 138, 36 links), mostly focusing on IL-6 (TLS = 147, 42 links) and IL-15 (TLS = 105, 39 links). The latter are firmly bound to inflammation processes, which also fall within the framework of the cluster (TLS = 224, 43 links).
***Cluster 2*** (yellow) focuses mainly on model organism research (animals [TLS = 50, 48 links], mice [TLS = 20, 45 links], and induced c57bl mice [TLS = 7, 41 links]), employing them in studies of signal transduction (TLS = 10, 38 links), high-fat diet (TLS = 16, 39 links), and physical conditioning research (TLS = 12, 37 links).
***Cluster 3*** (red) includes human studies according to sex and age differences concerning body composition (TLS = 21, 42 links) and BMI (TLS = 20, 43 links). Attention is directed towards insulin resistance (TLS = 39, 49 links), insulin (TLS = 11, 38 links), blood glucose (TLS = 12, 40 links), and identification of molecular biomarkers (TLS = 10, 39 links).
***Cluster 4*** (blue) is related to human energy metabolism (TLS = 15, 45 links), thermogenesis (TLS = 9, 29 links), and brown adipose tissue (TLS = 13, 42 links), and type 2 diabetes (TLS = 25, 47 links). It also involves myokines such as irisin (TLS = 30, 47 links) and fibronectins (TLS = 56, 49 links).

The summarization of keyword clusters from each of the decades are represented in Table 2.

**Table 2:** Changes in research subjects and topics in studies of obesity, myokines, and physical activity over 2 decades (2004–2013 and 2014–2024).

Clusters	Themes in clusters in first decade (2004–2013)	Themes in clusters in second decade (2014–2024)
Cluster 1	Analysis of signaling pathways.	Studies of muscle-fat interaction, including inflammation.
Cluster 2	Studies of skeletal muscles and model organisms; gene expression and energy metabolism.	Research of model organisms, using them to study signal transduction, high-fat diets, and physical conditioning.
Cluster 3	Human research focused on biomarkers (risk factors, adipokines [like leptin], intercellular signaling peptides [like fibronectins]), and complications of obesity (insulin resistance, inflammation, and metabolic syndrome).	Human research (according to sex and age differences) concerning body composition and BMI. Attention is directed towards insulin resistance, insulin, blood glucose, and the identification of molecular biomarkers.
Cluster 4	–	Human energy metabolism (including thermogenesis), brown adipose tissue, myokines (like irisin and fibronectins), and type 2 diabetes.

Furthermore, it was observed during the analysis that there are significant and frequently used keywords describing the study subjects (human according to sex [female, male] and age [adult, aged, youth, middle aged], and model organisms [mice, cultured cells]). Therefore, a separate bibliometric analysis was conducted to better understand which individual groups are most extensively analyzed and researched in this field and which of them yield the most insights regarding obesity, myokines, and exercise. In the first decade, obesity, myokines, and exercise were exclusively investigated in adult and elderly (both groups constitute 80% of age-specific keywords). Meanwhile in the second decade, younger subjects — middle-aged and even adolescents and young adults — are included in the studies (constituting 51% of age-specific keywords). Regarding sex, roughly equal attention is given to both male (57–58%) and female (42–43%) groups, with slightly more male participants in both decades.

In summary, based on the results of the bibliometric analysis, the topic of scientific interest in the context of obesity, myokines, and exercise in the second decade (2014–2024) was focused on molecular processes and new organokines (secreted by adipocytes and skeletal muscle) in the human body and the study of animal models.

## 4 Discussion

In recent years, an increasing number of studies have been conducted focusing on the epidemiology of obesity, predisposing factors, and prevention mechanisms. Obesity, characterized by an excess of white adipose tissue, disrupts metabolic equilibrium and contributes to chronic conditions including cardiovascular diseases, metabolic syndrome, and type 2 diabetes (T2D) (1, 4). It is known that exercise might act as a treatment helping to improve quality of life and reducing the risk of 26 different diseases, such as metabolic, cardiovascular, psychiatric, neurological, and pulmonary diseases; musculo-skeletal disorders; and cancer. For instance, in metabolic diseases like obesity, hyperlipidemia, metabolic syndrome, and T2D, a large volume of moderately intense aerobic exercise is recommended, ideally combined with strength training (at least 60 min of moderately intense activity daily). High-intensity exercise improves glycemic control more effectively than low-intensity exercise. Overall, it was found that long-term resistance and aerobic exercises are effective treatments in most cases of diseases (17, 18). However, the molecular mechanisms underlying obesity and exercise training or sedentary behavior (as external factors) and internal factors (such as myokines) are still not fully understood. Therefore, this study involved a bibliometric analysis of scientific publications and their data to identify and visualize research trends related to myokines, exercise, and obesity. The research results showed that obesity is currently a relevant research topic, and the molecular mechanism of muscle contraction is actively being investigated. From 2014 to 2024, ten times more scientific publications were published compared to the previous decade (2004–2013), with a 12.5-fold increase in the number of new topics (keywords) related to exercise, myokines, and obesity. The highest number of publications throughout the period was authored by Mantzoros et al. from the United States (7 publications).

Bibliometric analysis revealed that in 2004–2013 research on obesity and its complications (such as metabolic syndrome, T2D) focused on analysis of skeletal muscle, fibronectins, IL-6, and adipokines, as well as molecular processes related to insulin resistance and inflammation. Scientific studies demonstrated that the impact of cytokines on insulin sensitivity can be regulated through diet and exercise (2, 8). Over the most recent decade (2014–2024), researchers paid more attention not only to complications of obesity but also to the analysis of new signaling molecules such as myokines and adipokines. Moreover, attention has been given to adipose tissue, energy metabolism, and animal research, as well as a number of human molecular studies (according to sex and age differences) have also been conducted. Thus, in recent years, there has been progress in research related to obesity and exercise, with a focus on specific scientific topics. These studies take into account the complications of obesity, the development of those complications, and what gives positive health effects and could be used as a therapeutic preventive or treatment tool. Human studies and experiments with animal models have been conducted to address the molecular and physiological mechanisms of metabolism during exercise. The themes of metabolism, endocrine disorders, and complications of obesity have been widely discussed, along with analysis of the inflammatory process and the signaling molecules involved. Molecular studies have mainly focused on myokines, particularly irisin, and gene expression regulation (6, 19, 20). Myokines as mediators provide the conceptual basis for a whole new paradigm useful for understanding how skeletal muscle communicates with brain, liver, pancreas, adipose tissue and other organs and which myokines are related to health and/or participate in the pathogenesis of obesity.

### 4.1 Myokines: implications of physical exercise

It has been established that exercise and myokines production are closely related to the regulation of metabolism, stress response, and inflammation, and therefore they May play an important role in the pathogenesis of obesity and directly affect variables related to physical exercise and health (6, 16). For example, myokines such as myostatin, irisin, fibromodulin, and IL-6 are released during muscle contraction and May have anti-inflammatory effects, regulate metabolic processes, and positively influence the condition of the cardiovascular system (6, 7, 21, 22).

Below we introduce some common myokines that are most studied by researchers in the context of exercise and obesity. Some of the myokines still need more research.

#### 4.1.1 Interleukin-6

Myokine IL-6 functions as a pro-inflammatory cytokine (produced by immune cells) and an anti-inflammatory myokine (produced and secreted by contracting muscles). IL-6 was the first identified myokine and the most extensively studied. During acute, both aerobic and anaerobic exercise IL-6 concentration in blood serum increases (up to 100 times immediately after exercise), and its positive effects are observed throughout the body, including brain function, adipose tissue metabolism, energy metabolism, muscle regeneration, and more (7). IL-6 is reported to be able to induce glucose transporter type 4 (GLUT4) translocation to the cell membrane in response to insulin-stimulated glucose disposal and improve glucose tolerance (23). Although the mechanism underlying obesity-related IL-6 upregulation is not fully understood, increase in IL-6 levels affect redox balance, mitochondrial physiology, and satellite cells in skeletal muscle (7). Exercise increases IL-6 levels, especially in the presence of low muscle glycogen, suggesting a role for IL-6 in glucose disposal and improved tolerance. Some studies suggest that exercise duration is the main determinant of IL-6 release levels. Therefore, all intensity, both low, medium and high intensity aerobic-resistance combination exercise provides IL-6 levels change, but high-intensity combination exercise provides the most optimal improvement. Even 3 h after running an increase in the amount of IL-6 is observed (24–26). In essence, IL-6 has a therapeutic potential in the process of improving immune system, energy and glucose homeostasis in obesity through enhanced central IL-6 trans-signaling. IL-6 levels can be increased through exercise, especially high-intensity combination exercise, leading to improved glucose disposal and tolerance. Therefore, in the future IL-6 (such as selective inhibition of IL-6 trans-signaling) can be targeted as a potential therapeutic option for treating obesity and metabolic disorders (affecting adipose tissue and impairments of skeletal muscle glucose uptake) as well as for autoimmune and inflammatory conditions.

#### 4.1.2 Interleukin-15

Myokine IL-15 derived from skeletal muscles May reduce lipid accumulation in adipocytes, suggesting a role in exercise-mediated muscle-fat crosstalk (24). According to existing research, plasma IL-15 increases significantly in response to various exercise modalities (chronic combined aerobic exercise, resistance training, and short-term resistance training) and thereby plays a potential role in improving metabolism and regulating fat-lean body composition. Moreover, elevated circulating IL-15 levels has been shown to cause a significant decrease in body fat and prevent accumulation of adipose deposits resulting from high-fat diet. Similar results were observed in both human and animal (rodent) studies (11, 27–29). Furthermore, in human subjects acute exercise (even after 1 h) notably elevates plasma IL-15 levels and attenuates serum tumor necrosis factor *α* (TNF-α) and C-reactive protein levels, suggesting potential anti-inflammatory effects (30). This immuno-metabolic effects of IL-15 suggest it May be a potential therapeutic target for treating obesity.

#### 4.1.3 Follistatin-like 1 protein

The studies showed that Fstl1 is a myokine involved in muscle-adipose tissue crosstalk. Fstl1 facilitating lipid mobilization during and after various types but long-term exercise (11, 30). It was found that Fstl1 serum levels significantly increased during endurance exercise and through the recovery period, correlating with lipolysis and lean body mass (30). However, using transgenic animal models, FSTL1 has been implicated in multiple signaling pathways and its role during diseases like obesity remains unclear. Thus, Fstl1 has the potential to be used as a therapeutic target for treating obesity and improving metabolic health.

#### 4.1.4 Musclin

Musclin has been identified as an exercise-responsive myokine inducing animal models (mice) skeletal muscle mitochondrial biogenesis and abolishes muscle atrophy. Recent human and transgenic models’ studies suggest that musclin, an exercise-responsive myokine, has the ability to attenuate inflammation, oxidative stress, and apoptosis in skeletal muscles and cardiomyocytes under pathogenic conditions (31). While the potential benefits of musclin in the various physiological processes (muscle and/or cardiovascular systems) have been confirmed, its effects on lipid metabolism and obesity are not fully understood. Hence therapeutic potential of musclin is to be subject to further investigations.

#### 4.1.5 Irisin

The latest research object is irisin, which participates in metabolism, immune response, myogenesis, osteogenesis, neurogenesis, and central nervous system functioning. Irisin is a myokine, implicated in the pathogenesis of obesity-related complications, including dyslipidemia, T2D, and metabolic syndrome. Due to the diverse physiological effects of irisin on the body, it has attracted the interest of scientists worldwide and is widely studied in various health aspects, including the pathophysiology, treatment, and prevention of obesity (6, 26, 32–37). Myokine irisin, induced by chronic exercise, promotes brown fat-like development within white fat cells, activates thermogenesis, regulates oxidative stress and insulin resistance, thus, offering protection against obesity and diabetes (9, 10, 21, 26, 32, 33). Irisin concentration increases by ~15% immediately following an acute exercise, 8-week endurance training and resistance training in healthy adults. During and after exercise, thermogenesis and enhanced energy expenditure potentially improve metabolic parameters. Even short-term high intensity training increases cellular insulin sensitivity, and intensifies glucose and fat oxidation, particularly beneficial for individuals with obesity and T2D (34). Over the past decade, it has become clear that irisin is derived from the proteolytic processing of fibronectin type III domain-containing protein 5 (Fndc5). Studies have consistently found that serum irisin levels negatively correlate with 2 h plasma glucose, IHbA1c, and triglyceride levels (36, 37). Irisin also exhibits anti-atherosclerotic and neuroprotective properties. To mitigate cardiovascular risk, irisin has been suggested as a promising therapeutic target for addressing obesity and T2D (35). In rodent models, the administration of irisin improves insulin biosynthesis, promotes beta-cell functional mass accrual, enhances glycemic control, and facilitates weight loss, suggesting therapeutic potential in diabetic and obese individuals (19, 20, 36). Aerobic exercise improves glucose tolerance, possibly through the release of irisin and other various myokines (like interleukins), which enhance insulin sensitivity and regulate glucose and lipid metabolism (38). This suggests that irisin could be used as a therapeutic agent in diabetic and obese individuals to improve metabolic parameters and potentially mitigate cardiovascular risk.

### 4.2 Physical exercise and muscle-adipose tissue crosstalk

Current evidence indicates that myokines act to control adipose tissue functions, including lipolysis, browning, and inflammation, whereas adipokines mediate the beneficial actions of adipose tissue in the muscle, such as glucose uptake and metabolism (39–41). In the past decade, the number of scientific studies analyzing the structural and functional differences (types) of adipose tissue in more detail has increased. Our study showed that keywords used quite often are white adipose tissue, brown adipose tissue, and thermogenesis. White adipose tissue secretes adipocytokines, glucocorticoids, and sex hormones, whereas brown adipose tissue regulates body temperature through adaptive thermogenesis, controls triglyceride levels, stores glucose, and releases various adipocytokines and molecules such as prostaglandins, nitric oxide, and adipsin (3). Accumulation of ectopic fat, particularly visceral, cardiac, and muscle fat, occurs when adipocytes have already reached their maximal storage capacity. The enlargement of adipocytes triggers inflammation by disrupting the equilibrium between adipocytes and surrounding cells, particularly resident macrophages. This disruption leads to increased secretion of various inflammatory adipocytokines such as leptin, IL-6, TNF-*α*, angiotensinogen, adipsin, free fatty acids, and lactate, alongside a decrease in the levels of anti-inflammatory molecules like adiponectin (26, 30, 42). Adiponectin levels are reduced in obesity, hypertension, hyperlipidemia, T2D, and coronary atherosclerosis (43). Due to reduced plasma adiponectin levels observed in individuals with obesity, insulin resistance, or T2D, adiponectin is regarded as a biomarker for metabolic syndrome (4). The scientific publications showed that high intensity interval training (HIIT) is a time-efficient strategy to decrease fat-mass deposits, including those of abdominal and visceral fat mass. In recent studies, the most commonly used and most effective strategies are: both long-term (from 4 weeks to 6 months) and short-term (2 weeks) HIIT, as well as aerobic (running or cycling, intensity above 90% peak heart rate, high intensity work followed by recovery period), and eccentric resistance training (10 exercise in 3 sets of 10 repetitions in 80% of 1 maximum repetition (1RPM), which is followed by active rest of 15 repetitions of 20% of 1RPM), on average 3 sessions a week (44–46).

### 4.3 Pro-inflammatory state of obesity and anti-inflammatory responses to exercise

Excessive secretion of pro-inflammatory adipocytokines from adipocytes and macrophages within adipose tissue contributes to low-grade systemic inflammation in individuals with obesity (47, 48, 54). According to existing research, obesity induces chronic low-grade inflammation, marked by increased secretion of inflammatory molecules like TNF-*α*, IL-6, and monocyte chemoattractant protein-1 (MCP-1), primarily by visceral adipose tissue (49). Inflammatory processes cause tissue dysfunction, hypoxia, and injury (3). Regular (chronic) exercise (>2 weeks, both aerobic or resistance training) offers protective benefits against the harmful effects of pro-inflammatory adipokines through the secretion of muscle-derived myokines adiponectin, omentin, vaspin (47). These myokines counteract the pro-inflammatory effects of adipokines, contributing to systemic anti-inflammatory responses following exercise. However, most adults with chronic non-communicable diseases (like obesity) fail to meet recommended exercise levels (38). Recent studies indicated that exercise-training intervention could induce myokines (especially irisin) secretion, providing the potential for treatment of obesity. Overall, understanding the molecular properties of obesity and exercise can help develop future treatment strategies to improve health and prevent obesity.

### 4.4 Physical exercise and obesity-related metabolic complications

Since the beginning of the obesity and myokine research era, much attention is paid to the pathophysiology of metabolic syndrome, T2D, insulin sensitivity disorders, endocrine disorders and the influence of exercise on changes in metabolic health indicators. Skeletal muscle insulin resistance significantly impacts whole-body metabolism, as it is responsible for the majority of postprandial glucose uptake (80%). Obesity and insulin resistance at this site are a substantial contributor to the development of T2D (30). In T2D and an insulin-resistant state, pancreatic *β*-cells compensate by enhancing their functionality and mass trying to maintain normal blood glucose levels (euglycemia), but eventually, they undergo dysfunction and partial loss (4). Insulin plays a crucial role in regulating glucose levels by suppressing hepatic glucose production and promoting glucose storage as glycogen (50). However, in T2D, insulin fails to regulate glycogen synthesis and glucose production, leading to increased hepatic gluconeogenesis and hyperglycemia (50). The combination of hyperglycemia and dyslipidemia accelerates *β*-cell death, further reducing insulin secretion and exacerbating hyperglycemia. Moreover, accumulation of visceral fat and insulin resistance results in increased delivery of fatty acids to the liver, leading to elevated triglyceride synthesis and a heightened risk of coronary artery disease in individuals with hypertriglyceridemia (3, 50). It has been shown that exercise normalizes non-physiological serum insulin and glucose levels (3, 5, 15, 51). Exercise May stimulate the synthesis and release of different myokines, increase insulin-like growth factor 1 (IGF-1) levels, however, decrease IL-6 and IL-15 levels in patients with T2D, which controversially potentially improving metabolic health in case of T2D and in individuals with obesity or metabolic syndrome. In diabetic and/or obese animal models, irisin May enhance insulin biosynthesis, promote an increase of beta-cell functional mass, improve glycemic control, and according to some research induces weight loss. In rodent models, the administration of irisin improves insulin biosynthesis both 24 h after eccentric resistance training program (6 weeks, 3 times a week) and 24 h after 1 session of eccentric resistance exercise consisting of 8 repetitions descending from a ladder with a slope of 80 degree, with an extra load of two times body weight (100% 1RM) (19, 20). Based on previous human research results, high-intensity (above 90% peak heart rate) training is more successful in reducing whole body adiposity, while lower intensities have a greater effect on changes in abdominal and visceral fat mass (44). But data on chronic exercise are less conclusive (36, 37, 52, 53).

The present bibliometric analysis is a reliable and global source of knowledge that allows us to properly understand the advances in research related to obesity and physical exercise. However, the present study had a few limitations. First, although PubMed was considered a reliable database source for conducting bibliometric analysis, the data in the present study might not be comprehensive without adding other databases (e.g., Web of Science, Embase, Medline, and Scopus). Therefore, future research related to myokines and exercise in the context of obesity should extend this bibliometric analysis to other databases. Second, we only selected articles published in English, thereby resulting in language bias. However, results of this study provide insight into trends in research of molecular mechanisms of obesity and benefits of exercise, and presented the need for further development in these topics.

## 5 Conclusion

The relationship between myokines and exercise in the context of obesity has been a widely researched topic in recent years, and the number of global publications is growing exponentially. The increasing trend of publications indicates that the molecular mechanism of the human body is one of the most relevant topics related to obesity and exercise. In addition to obesity, its complications such as inflammation, insulin resistance, and type 2 diabetes are commonly investigated. The results highlight the importance of myokines in modulating metabolic processes and the potential therapeutic implications of exercise in managing non-communicable diseases such as obesity and type 2 diabetes. It has been observed that studies with animal models and cell culture focusing on molecular processes and specific signaling pathways are also relevant. In general, over the past decade, significant attention has been given to the search for biomarkers and the analysis of signaling molecules released during muscle contraction. Irisin, as a newly identified myokine responsive to physical exercise, has emerged as a promising marker for the prevention and control of obesity. The interaction between myokines and adipokines holds significant promise for developing novel therapeutic strategies for obesity, diabetes, and conditions linked to muscle atrophy. Understanding how these signaling molecules communicate could lead to innovative treatments targeting these prevalent metabolic disorders. Future therapeutic approaches that focus on managing insulin resistance and regular physical exercise can produce anti-inflammatory effects. Such strategies could not only enhance metabolic health but also mitigate inflammation, further improving clinical outcomes for patients. Future research should continue exploring these areas, leveraging advances in molecular biology to uncover new therapeutic targets and strategies for managing obesity.

## Data Availability

The original contributions presented in the study are included in the article/supplementary material, further inquiries can be directed to the corresponding author.
